# Immunogenicity and Protection From a Single Dose of Internationally Available Killed Oral Cholera Vaccine: A Systematic Review and Metaanalysis

**DOI:** 10.1093/cid/cix1039

**Published:** 2017-11-21

**Authors:** Anna Lena Lopez, Jacqueline Deen, Andrew S Azman, Francisco J Luquero, Suman Kanungo, Shanta Dutta, Lorenz von Seidlein, David A Sack

**Affiliations:** 1 Institute of Child Health and Human Development, National Institutes of Health, University of the Philippines, Manila; 2 Delivering Oral Vaccine Effectively, Department of International Health, Johns Hopkins University, Baltimore, Maryland; 3 Department of Epidemiology, Johns Hopkins University, Baltimore, Maryland; 4 Epicentre, Paris, France; 5 National Institute of Cholera and Enteric Diseases, Beliaghata, Kolkata, West Bengal, India; 6 Mahidol-Oxford Tropical Medicine Research Unit, Faculty of Tropical Medicine, Mahidol University, Bangkok, Thailand

**Keywords:** cholera, oral cholera vaccine, cholera vaccine, *Vibrio cholera*

## Abstract

In addition to improved water supply and sanitation, the 2-dose killed oral cholera vaccine (OCV) is an important tool for the prevention and control of cholera. We aimed to document the immunogenicity and protection (efficacy and effectiveness) conferred by a single OCV dose against cholera. The metaanalysis showed that an estimated 73% and 77% of individuals seroconverted to the Ogawa and Inaba serotypes, respectively, after an OCV first dose. The estimates of single-dose vaccine protection from available studies are 87% at 2 months decreasing to 33% at 2 years. Current immunologic and clinical data suggest that protection conferred by a single dose of killed OCV may be sufficient to reduce short-term risk in outbreaks or other high-risk settings, which may be especially useful when vaccine supply is limited. However, until more data suggest otherwise, a second dose should be given as soon as circumstances allow to ensure robust protection.

## Teaser

Current immunologic and clinical data suggest that protection conferred by a single dose of killed oral cholera vaccine may be sufficient to reduce short-term risk in outbreaks or other high-risk settings, which may be especially useful when vaccine supply is limited.

Cholera is an acute watery diarrheal disease that can spread rapidly and lead to widespread outbreaks. An estimated 2.9 million cholera cases occur annually in endemic countries [1]. Improved water quality, sanitation, and hygiene (WASH) are the cornerstone for cholera prevention and control, but the world is falling short of meeting these targets [2]. Displacements due to natural disasters and conflicts and population growth will result in continuing cholera outbreaks in the future unless preventive measures are applied.

In parallel with WASH, timely treatment, and community engagement, the World Health Organization (WHO) recommends that oral cholera vaccination be considered in areas where the disease is endemic, as part of the response to outbreaks, or in a humanitarian crisis where there is a high risk of cholera [3]. There are 3 internationally available, killed oral cholera vaccines (OCV). The first is a monovalent (*Vibrio cholerae* O1) whole-cell OCV that contains the recombinant B subunit, marketed as Dukoral (Valneva, Lyon, France). Randomized, placebo-controlled trials of earlier versions of Dukoral in Bangladesh showed a 2-dose protective efficacy at 1 and 3 years of follow-up of 74% [4] and 64% [5], respectively. Dukoral was the first OCV that was internationally licensed in 1991 and WHO prequalified in 2001, but it is relatively expensive and requires a buffer for administration. Dukoral is primarily used by travellers.

The second vaccine is Shanchol (Shantha Biotechnics Ltd, Hyderabad, India), a bivalent (*V. cholerae* O1 and O139) whole-cell OCV. A randomized, placebo-controlled trial in India showed that a 2-dose regimen confers 67% protective efficacy against cholera within 2 years of vaccination [6], 66% at 3 years [7], and 65% at 5 years [8]. Continued protection up to 5 years in this endemic setting may have been due to boosting from natural exposure. Shanchol was licensed in India in 2009 and received prequalification from WHO in 2011. WHO and its partners established an OCV stockpile [9]. From 2013 to 2017, over 25 million OCV doses have been requested from the stockpile, of which 71% were approved and 51% were shipped to countries for 46 deployments [10]. The high demand has been ascribed to the growing evidence of mass OCV campaigns’ feasibility, effectiveness and complementarity with WASH interventions [11].

A third vaccine is Euvichol (Eubiologics, Gangwon-do, South Korea), another bivalent OCV based on the same formulation as Shanchol. At the time of writing, there is no published clinical efficacy data for Euvichol. However, following a phase 1 trial in Korea [12] and a bridging noninferiority immunogenicity study in the Philippines [13], Euvichol was licensed and WHO prequalified in 2016. WHO prequalification is necessary for the purchase of vaccines by UN agencies, including UNICEF.

Two-dose regimens for Dukoral, Shanchol, and Euvichol are recommended; however, delivery of 2 doses can be challenging during emergency situations. The difficulties include accessing the same population twice, maintaining vaccine storage, and retaining vaccination staff during the inter-dose period. Also, the response lag combined with the shortened duration of outbreaks after a first dose is given may render the additional protection conferred by a second dose less important. Previous modeling suggests that reactive vaccination campaigns that use a single dose of OCV may prevent more cases and deaths than a 2-dose campaign when vaccine supplies are limited, while at the same time reducing logistical complexity [14]. Our primary question is how well a single-dose regimen of killed OCV protects against cholera. As there are relatively few studies that document OCV efficacy and effectiveness in conferring protection against disease and since a vaccine-induced increase in vibriocidal antibody titer has been linked with protection [15], we included both a systematic review of the efficacy and effectiveness data and a systematic review and metaanalysis of a larger body of immunologic response data.

## METHODS

The systematic review and metaanalysis were conducted according to preferred reporting items for systematic review and meta-analyses guidelines [16]. We searched PubMed, the Cochrane Central Register of Controlled Trials, and Scopus from 1 January 2005 to 13 November 2016 combining MeSH and free-text terms for the following: “killed cholera vaccine,” “oral cholera vaccine,” OCV, immun,* serolog,* “immune response,” “serologic response,” vibriocidal, protect,* efficac,* and effective,* without any language or age restrictions. The search was limited to the past decade, from 2005 onward, to include only studies on the currently available vaccine formulations (Dukoral underwent formulation changes before international licensure and WHO prequalification). We also contacted public health personnel and experts in the field to identify unpublished documents such as meeting presentations to ensure completeness. The detailed search strategy is shown in Supplementary Figure S1.

Titles and abstracts were compiled in Endnote X6 (Thomson Reuters, Philadelphia, Pennsylvania). Two authors (A. L. L. and J. D.) screened the list of titles and abstracts independently to ensure that they included information on immunogenicity as measured by vibriocidal antibodies or protection following 1 dose of current formulations of internationally available killed OCV (Dukoral, Shanchol, and Euvichol). Case reports, animal studies, sociobehavioral studies, economic evaluations, and articles that did not report original research (eg, comparison of previously reported data, reviews, modeling studies, correspondence, and editorials) were excluded. The full texts of eligible articles were downloaded and reviewed in detail. Immunogenicity and vaccine protection (efficacy or effectiveness) data were extracted and analyzed. Basic analysis and data summarization were done using Microsoft Excel 2011 (Seattle, Washington). We used the GRADE guidelines to assess the risk of bias [17] and the quality of evidence [18] of the included articles, as shown in the Supplementary Table S1.

### Immunogenicity

From the reports on vaccine immunogenicity, we tabulated the number and age group of participants, study location, vibriocidal baseline geometric titers, geometric mean fold rises (GMF rise) after a first and subsequent vaccine dose, and the number and percentage of participants who seroconverted after a first and subsequent dose. Following convention, seroconversion was defined as a ≥4-fold rise from baseline. Vibriocidal responses to Inaba and Ogawa were tabulated separately. Vibriocidal responses to O139 were not included in this analysis because of the variability in the laboratory testing among different groups and because its utility as an immunologic correlate of protection remains unknown [19]. Furthermore, outbreaks of cholera due to *V. cholerae* O139 have not been reported during the past decade, although sporadic cases have been identified [20]. We performed a metaanalysis of the proportion of individuals who seroconverted after 1 and 2 doses of OCV using binomial-normal random effects regression models [21], including baseline geometric mean titer (GMT) as a covariate. The metaanalysis focused on the bivalent vaccines (Shanchol and Euvichol) since they are currently the most commonly used OCVs and because of the sparse data available on Dukoral. GMTs were centered at the mean and scaled (by the standard deviation); the pooled seroconversion estimates that were presented assumed the mean GMT value. Three age groups were modeled separately for each serotype, in addition to models that combined all age groups. Studies varied in the reporting of results by age groups; for the purpose of the metaanalysis, we classified results from participants aged ≥15 years (including those ≥18 years) as from “adults,” those from participants aged <18 years as from “children,” and those from participants aged ≤5 years as from “young children.” We estimated the I^2^ statistic as a measure of unexplained heterogeneity between studies. Metaanalyses were performed with the metafor package in R (version 3.2.3); data used are available at https://github.com/scottyaz/singledose-immuno-review.

### Vaccine Protection

We included studies on killed OCV efficacy and effectiveness against cholera. We defined vaccine efficacy as the protection conferred under ideal conditions of a randomized, controlled trial, whereas vaccine effectiveness is the protection when the vaccine is given under actual public health situations, assessed by observational studies. From the reports on vaccine protection, we tabulated data on study design, site and year, intervention, study population and vaccine coverage, primary assessment of protection (clinical endpoints and definitions), total number of cholera cases, main infection serotypes and biotypes, estimated vaccine efficacy or effectiveness after a single dose with subanalysis by disease severity or age group, and duration of follow-up. We plotted the estimated vaccine efficacy or effectiveness by duration of follow-up.

## RESULTS

**Figure 1.**
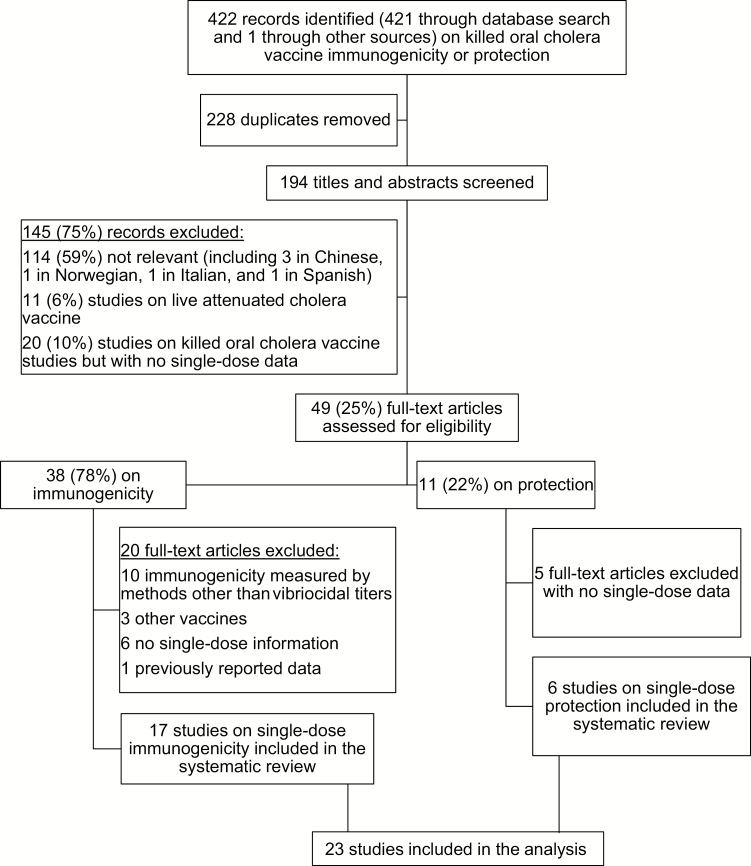
Selection of reports included in the analysis.

We identified 422 records on killed OCV immunogenicity or protection, 421 through the database search and 1 meeting presentation (Figure 1). We removed 228 duplicates and screened the titles and abstracts of 194 articles, of which 145 (75%) were excluded and 49 (25%) full-text articles were downloaded and reviewed. Of these, 23 studies fulfilled the inclusion criteria and were included in the systematic review: 17 articles and 1 presentation on single-dose vaccine immunogenicity and 6 articles on single-dose vaccine protection (see references listed in Supplementary Data). General descriptions, risk of bias within the study [17], main biases, and quality grading scores [18] of the 23 included studies are shown in Supplementary Table S1.

### Immunogenicity

Four articles reported on the immunogenicity of the monovalent OCV that contains the recombinant B subunit (Dukoral), while 13 reported on the bivalent OCV, Shanchol, and Euvichol (see references listed in Supplementary Data). Two articles reported on the persistence of antibodies up to 1 year following 2 doses of the monovalent vaccine (see references listed in Supplementary Data). The baseline titers and vibriocidal immune response to Ogawa and Inaba serotypes following killed oral cholera vaccination are shown in Tables 1 and 2, respectively. Baseline titers varied considerably by age group and across the study sites, with the highest in Kolkata, India, and the lowest in Chungnam, Korea. We found that 43%–95% of participants seroconverted to the Ogawa serotype and 52%–90% to the Inaba serotype following the first dose.

**Table 1.** Vibriocidal Immune Response to Ogawa Serotype Following Killed Oral Cholera Vaccination

Study	Number of Participants (Age Group)	Study Site	Baseline Geometric Mean Titers	Interval Between Doses, days	No. (%) Who Seroconverted After:		Geometric Mean Fold Rise After:	
					1st Dose^a^	2nd Dose^a^	1st Dose^a^	2nd Dose^a^
Monovalent OCV that contains the recombinant B subunit								
Ahmed 2009	49 (10–18 months)	Dhaka, Bangladesh	NA	14	NA	(56)	4	5
	47 (6–9 months)			14	NA	(57)	2	5
Alam 2011	30 adult 1-dose recipients	Dhaka, Bangladesh	27	NA	(50)^b^	NA^c^	5-fold by day 3^b^	5-fold by day 17^d^
	30 adult 2-dose recipients		26	14	(43)^b^	(36)^d^	5-fold by day 3^b^	5-fold by day 17^d^
Leung 2012	20 children aged 2–5 years	Dhaka, Bangladesh	37	14	0	(65)	2-fold by day 3^b^	23-fold by day 7^e^
	20 children aged 6–17 years		32	14	(35)	(90)	3-fold by day 3^b^	42-fold by day 7^e^
Bivalent OCV								
Saha 2011	53 (18–45 years)	Dhaka, Bangladesh	61	14	(70)^e^	(59)^e^	7^e^	NA
	55 (2–4 years)		39		(75)^e^	(75)^e^	9^e^	NA
	54 (12–24 months)		9		(78)^e^	(74)^e^	6^e^	NA
Charles 2014	23 adults	Haiti	14	14	(77) ^e^	(91)^e^	19^e^	19 ^e^
	45 (6 to 17 years)		21		(69) ^e^	(74)^e^	11^e^	10 ^e^
	42 (1–4 years)		14		(64) ^e^	(73)^e^	9^e^	10 ^e^
Kanungo 2015	96 (15+ years)	Kolkata, India	329	14	57 (59)	51 (53)	6	5
	90 (6–14 years)		102	14	79 (88)	65 (66)	22	13
	91 (15+ years)^f^		475	14	50 (55)	37 (41)	4	3
	93 (6–14 years)^f^		236	14	65 (70)	67 (72)	16	9
Kanungo 2015	86 adults (≥18 years)	Kolkata, India	364	14	48 (56)	39 (45)	6	4
			359	28	52 (62)	41 (49)	6	4
	83 children (1–17 years)		125	14	63 (75)	61 (73)	19	11
			131	28	65 (79)	59 (72)	16	8
Aloysia 2015	112 (15+ years)	Philippines	69	14	(78)	(69)	14	11
	112 (5–14 years)		18		(86)	(88)	48	43
	112 (1–4 years)		3		(72)	(96)	61	82
Desai 2015	37 (18+ years)	Ethiopia	24	14	24 (65)	26 (70)	13	13
	45 (1–17 years)		4		36 (80)	38 (84)	35	35
Ivers 2015	25 (HIV+ adults) 25 (HIV– adults)	Haiti	11 14	14	(52)^e^ (77)^e^	(65)^e^ (91)^e^	6^e^ 10^e^	7 ^e^ 13 ^e^

Baik 2014	25 adults	Korea	4	14	19 (95)	19 (95)	115	108
Baik 2015	Shanchol 376 adults 235 (1–17 years)	Philippines	74	14	295 (79)	278 (74)	17	13
			13		197 (84)	207 (88)	49	57
	Euvichol 377 adults 231 (1–17 years)		77		322 (85)	302 (80)	22	16
			13		200 (87)	209 (90)	61	66
Saha 2016	143 adults^g^	Bangladesh	71	14	106 (74)^e^	103 (72)^e^	NA	7^e^
Iyer 2016	37 (1–5 years)^h^	South Sudan	15	~21	9 (82)	11 (79)	11	14
	67 (6–17 years) ^h^		28		8 (53)	18 (55)	4	4
	101 (18–59 years)^h^		36		20 (43)	28 (52)	7	5
Matias 2016	22 adults	Haiti	35	14	14 (64)	16 (76)	7	5

See References Listed in Supplementary Data.

Abbreviations: HIV, human immunodeficiency virus; NA, not available; OCV, oral cholera vaccine.

a Blood for vibriocidal tests was obtained at 14 days after said dose, unless otherwise specified.

b Blood for vibriocidal tests was obtained at 3 days after said dose.

c Result of vibriocidal tests on 30th day after the single dose: 44%.

d Blood for vibriocidal tests was obtained on 16th day after the said dose or 30 days after the first dose.

e Blood for vibriocidal tests was obtained on 7th day after said dose, ie, 7 or 21 days after the first dose.

f These individuals had received vaccine 5 years earlier.

g Results included are only for those who received vaccine at the current storage recommendation of 2°C –8°C.

h Not all individuals were sampled at both time points to denominators for seroconversion changes.

**Table 2.** Vibriocidal Immune Responses to Inaba Serotype Following Killed Oral Cholera Vaccination

Study	Number of Participants (Age Group)	Study Site	Baseline Geometric Mean Titers	Interval Between Doses, days	No. (%) Who Seroconverted After:		Geometric Mean Fold Rise After:	
					1st Dose^a^	2nd Dose^a^	1st Dose^a^	2nd Dose^a^
Bivalent oral cholera vaccine								
Kanungo, 2009	37 (18+ years)	India	186	14 days	24 (65)	17 (46)	9	5
	39 (1–17 years)		37	14 days	34 (87)	32 (82)	47	24
Saha, 2011	53 (18–45 years)	Bangladesh	55	14 days	(60)^f^	(57)^c^	9 ^c^	NA^b^
	55 (2–4 years)		55		(78)^f^	(76) ^c^	12 ^c^	NA^b^
	54 (12–24 months)		8		(52)^f^	(72) ^c^	7 ^c^	NA^b^
Charles, 2014	23 adults	Haiti	11	14 days	(77) ^f^	(91) ^c^	19 ^c^	19 ^c^
	45 (6 to 17 years)		27		(69) ^f^	(74) ^c^	11 ^c^	10 ^c^
	42 (1–4 years)		16		(64) ^f^	(73) ^c^	9 ^c^	10 ^c^
Kanungo, 2015	96 (15+ years)	India	171	14 days	67 (70)	58 (60)	7	5
	90 (6–14 years)		50	14 days	(88)	(79)	26	14
	91 (15+ years)		238	14 days	(57)	(51)	5	4
	93 (6–14 years)		81	14 days	(85)	(82)	33	16
Kanungo, 2015	86 adults (≥18 years)	India	191	14 days	59 (69)	47 (55)	7	5
			144	28 days	55 (66)	49 (58)	9	5
	84 children (1–17 years)		47	14 days	72 (86)	67 (80)	30	18
			89	28 days	73 (89)	63 (77)	21	11
Aloysia, 2015	112 (15+ years)	Philippines	36	14 days	(83)	(78)	25	18
	112 (5–14 years)		3		(88)	(87)	58	49
	112 (1–4 years)		1		(88)	(89)	67	67
Desai, 2015	54 (18+ years)	Ethiopia	16	14 days	37 (70)	43 (81)	11	15
	53 (1–17 years)		6		39 (74)	41 (77)	13	13
Ivers, 2015	25 HIV+ adults	Haiti	11	14 days	(65)^c^	(74) ^c^	7 ^c^	7 ^c^
	25 HIV– adults		11		(82) ^f^	(91) ^c^	17 ^c^	20^c^
Baik, 2014	20 adults	Korea	4	14 days	18 (90)	19 (95)	74	94
Baik, 2015	Shanchol 376 adults 235 (1–17 years)	Philippines	36	14 days	315 (84)	287 (76)	30	21
			12		198 (84)	209 (89)	51	52
	Euvichol 366 adults 236 (1–17 years)		36		317(84)	308 (82)	32	22
			12		198 (86)	202 (87)	55	51
Saha, 2016	143 adults^d^	Bangladesh	99	14 days	109 (76)^c^	105 (73)^c^	11^c^	9^c^
Iyer, 2016	37 (1–5 years) 67 (6–17 years) 101 adults (18–59 years)	South Sudan	11	~21 days	9 (75)	12 (80)	11	9
			30		8 (53)	12 (38)	2	3
			22		28 (61)	31 (57)	8	7
Matias, 2016	22 adults	Haiti	29	14 days	16 (73)^c^	17 (81)^c^	9^c^	9^c^

See References Listed in Supplementary Data.

Abbreviation: HIV, human immunodeficiency virus.

a Blood for vibriocidal tests was obtained at 14 days after said dose, unless otherwise specified.

b Not available.

c Blood for vibriocidal tests was obtained on 7th day after said dose, ie, 7 and 21 days after the first dose.

d Results included are only for those who received vaccine at the current storage recommendation of 2°C–8°C.

**Figure 2.**
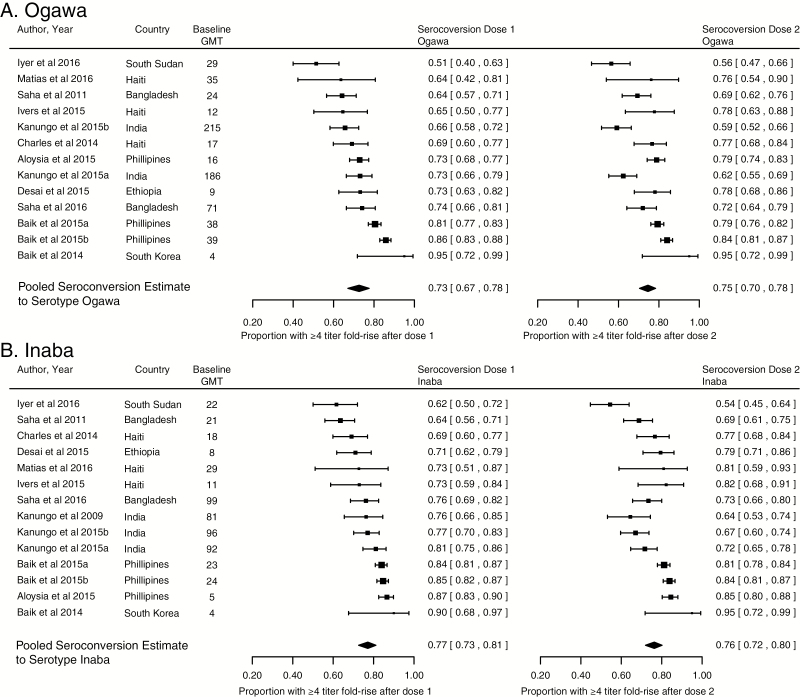
Seroconversion after the first and second dose of a bivalent oral cholera vaccine, all age groups. Abbreviation: GMT, geometric mean titer.

**Figure 3.**
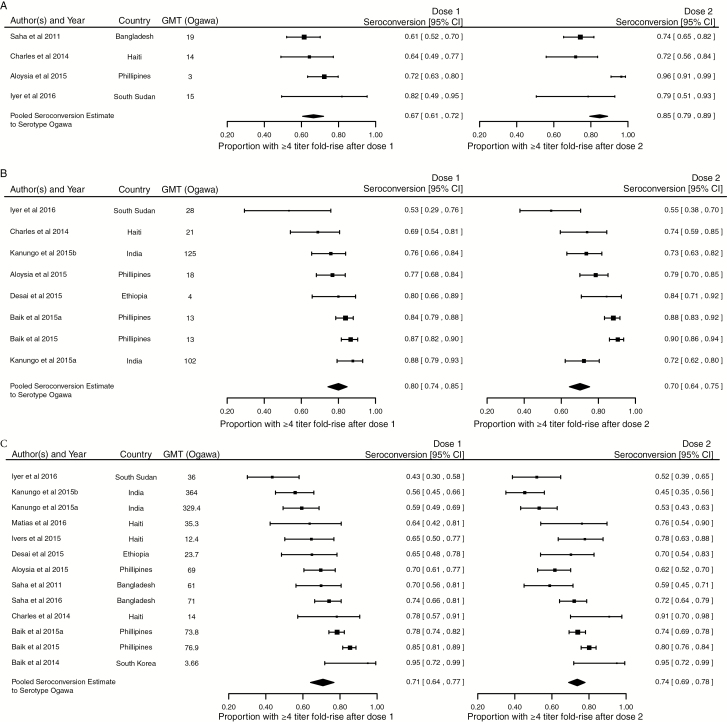
Seroconversion to the Ogawa serotype after the first and second dose of a bivalent oral cholera vaccine in young children *(A*), children *(B*), and adults *(C*). Abbreviations: CI, confidence interval; GMT, geometric mean titer.

**Figure 4.**
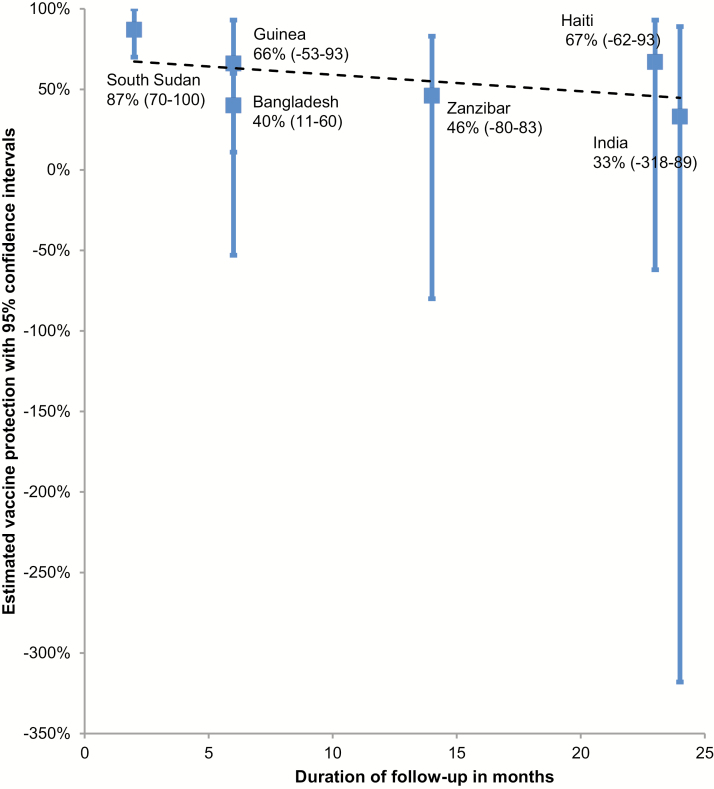
Estimated protection (95% confidence intervals) conferred by a single dose of killed oral cholera vaccine, by study site and duration.

The results of the metaanalysis are shown in Figures 2, 3, and 4. Overall, the median proportion of individuals who seroconverted after the first dose of a bivalent OCV was 73% (95% confidence interval [CI], 67%–78%; I^2^ = 85.4%) to the Ogawa serotype and 77% (95% CI, 73–81%; I^2^ = 64.3%) to the Inaba serotype (Figure 2). In the subanalysis of study populations with young children, children, and adults, the median proportion of individuals who seroconverted to Ogawa after the first dose was 67% (95% CI, 61%–72%), 80% (95% CI, 74%–85%), and 71% (95% CI, 64%–77%), respectively (Figure 3A, B, and C). The responses by age group to the Inaba serotype were consistent with that to the Ogawa serotype (Supplementary Figure S2*A*, *B*, and *C*).

Overall, in adults and children, a second dose of OCV did not increase the median seroconversion compared to the response to the first dose (Figures 2, 3C, and 3B). However, in the analysis limited to young children (Figure 3A), the proportion who seroconverted to Ogawa increased from 67% (95% CI, 61%–72%; I^2^ = 0) after dose 1 to 85% (95% CI, 79%–89%; I^2^ = 0) after dose 2 (*P* = .002 for difference after adjusting for baseline GMT), with a smaller and statistically insignificant increase for Inaba (*P* = .12; Supplementary Figure S2A).

Only 1 article reported on the immunogenicity among infants (using the monovalent OCV that contains the recombinant B subunit; Dukoral) [22], which showed a significantly higher GMF rise after the second dose only among those in the 6- to 9-month age group and not in the 10- to 18-month age group. One report on the immune response 5 years after a 2-dose vaccination schedule [23] showed no significant differences in baseline titers among those previously vaccinated compared to those who had not received the vaccine in the past.

Alam et al compared the vibriocidal responses of 1- and 2-dose recipients to 70 adult cholera patients [24]. Adult cholera patients had a 96 GMF rise from baseline (considered as the second day of presentation) in vibriocidal response to the homologous serotype as the infecting organism, and 93% of patients had a ≥4-fold rise from baseline 30 days later. Vibriocidal titers gradually declined until at 1 year of follow-up when 43% still had ≥4-fold titer from baseline. In comparison, 43%–50% of vaccinated adults seroconverted (≥4-fold titer from baseline) after the first dose with a 5-fold GMF rise by day 3 (Table 1). Leung et al reported on the vibriocidal responses of comparably aged pediatric patients [25]. Cholera patients who were aged 2–5 years had the highest GMF rise from baseline on the seventh day of presentation. Although increases from baseline were comparable in all age groups, vibriocidal antibodies waned earlier and were at baseline levels on the 42nd or 90th day after the first dose [26].

### Vaccine Protection

We identified 1 randomized study and 5 observational studies that reported on single dose protection (see references listed in Supplementary Data). The study design, site and year, intervention, study population and vaccine coverage, primary assessment of protection (clinical endpoints and definitions), total number of cholera cases, main infection serotypes and biotypes, estimated vaccine efficacy or effectiveness after a single dose with subanalysis by diseases severity or age group (when available), and duration of follow-up are shown in Table 3. There was 1 study on the monovalent OCV that contains the recombinant B subunit (Dukoral) [27], while 5 reported on bivalent OCV (Shanchol) (see references listed in Supplementary Data). A single dose of killed OCV conferred 87% protection at 2 months, declining to 33% at 2 years of follow-up (Table 3 and Figure 4). Only 1 study assessed vaccine protection by age group; the single-dose protection in young children was considerably lower than in older children and adults [28].

**Table 3.** Estimated Protection Conferred by a Single Dose of Killed Oral Cholera Vaccine

Study	Site (Study Year), Study Design	Vaccine	Study Population and Number of Participants	Primary Assessment of Protection (Clinical Endpoints and Definitions)	Total Number of Cholera Cases and Serotypes and Biotypes	Vaccine Protection after 1 Dose (95% Confidence Interval)	Duration of Follow-up (Months)
Wierzba, 2015	Odisha, India (2011), test-negative design	Bivalent OCV (Shanchol)	Of 51488 eligible residents of the study area, 31552 (61%) received at least 1 dose and 23751 (46%) received 2 doses	Compare odds of having been vaccinated between cholera cases and test- negative controls • Cases were diarrhea patients found positive for *Vibrio cholerae* • Controls were diarrhea patients negative for *V. cholerae* infection	44 patients included in the analysis • 44 (100%) O1 Ogawa, of which 34 (77%) were El Tor variant and 10 (23%) were hybrid (El Tor/classical) biotypes	• 33% (−318 to 89)	24
Ivers, 2015	Haiti (2012), case-control design with bias-indicator study	Bivalent OCV (Shanchol)	45417 people were vaccinated in the campaign, 91% of whom received both doses	Compare odds of having been vaccinated between cholera cases and matched controls • Cases were diarrhea patients with a stool sample positive for *V. cholerae* O1 • Controls were individuals who did not seek treatment for diarrhea between the first day of study enrollment and the date of onset of symptoms in their corresponding case, matched to each case by location of residence, enrollment time (within 2 weeks of the case), and age group (1–4 years, 5–15 years, and >15 years)	48 patients (1 excluded from the analysis due to lost data) • 36 (37%) O1 Ogawa • 11 (23%) O1 Inaba	• 67% (–62 to 93)	23
Khatib, 2012 [27]	Zanzibar (2008), cohort design with bias indicator study	Monovalent OCV that contains the recombinant B subunit (Dukoral)	Of 48178 eligible residents of the study area, 23921 (50%) received 2 complete doses of vaccine	Compare incidence of cholera in recipients of the vaccine and nonrecipients	42 patients included in the primary analysis • 42 (100%) O1 El Tor Ogawa	• 46% (–80 to 83)	14
Luquero, 2014	Boffa and Forecariah, Guinea (2012), case-control design with bias-indicator study	Bivalent OCV (Shanchol)	Target population was163000 people in Boffa district and 46000 people in parts of Forecariah; coverage with at least 1 dose was 92% in Boffa and 71% in Forecariah	Compare odds of having been vaccinated between cholera cases and matched controls • Cases were diarrhea patients with a stool sample positive for *V. cholerae* O1 • Controls were neighbors of the case who did not seek treatment for diarrhea between the first day of study enrollment and the date of onset of symptoms in their corresponding case, matched to each case by age group (1–4, 5–9, 10–19, 20–29, 30–39, or ≥40 years)	40 patients included in the primary analysis; of the 36 for whom a specimen was sent for culture and PCR analysis: • 18 (50%) O1 El Tor Ogawa; • 13 had positive results of culture and PCR • 5 had positive PCR results but negative culture results	• 66% (−53 to 93) based on culture or PCR positive cholera • 43% (−84 to 82) based on RDT positive cholera	6
Qadri, 2016 [28]	Bangladesh (2013), randomized, control trial	Bivalent OCV (Shanchol)	204700 persons underwent randomization, received 1 dose, and were included in the analysis (102552 received vaccine and 102148 received placebo)	Compare incidence of cholera in randomly assigned recipients of vaccine and placebo	101 cholera cases included in the analysis: • 100 (99%) O1 El Tor Ogawa • 1 (1%) O1 El Tor Inaba	• 40% (11 to 60) against all cholera episodes • 63% (24–82) against severely dehydrating cholera • 56% (16 to 77), 63% (−39 to 90), and 16% (49 to 53) against all cholera episode among persons vaccinated at the age of ≥15 years, 5–14 years, and 1–4 years, respectively	6
Azman, 2016 [34]	Juba, South Sudan (2015), case-cohort study	Bivalent OCV (Shanchol)	Juba was estimated to have between 500 000 and 1 million inhabitants with massive population movements because of civil strife; 140249 doses were administered in targeted areas of Juba [11]	Compare hazard ratios of cholera between unvaccinated and vaccinated persons	34 cholera cases included in the analysis	• 87% (70–100)	2

See References Listed in Supplementary Data.

Abbreviations: OCV, oral cholera vaccine; PCR, polymerase chain reaction; RDT, rapid diagnostic test.

## DISCUSSION

We found a significant immunologic response to a single dose of killed OCV. Following a titer rise post–first dose, there was little change after a subsequent dose when given within 14–28 weeks after the first. The exception was in young children where a second dose substantially increased the proportion who seroconverted to the Ogawa serotype (*P* = .002). The OCV immune response correlated with the evidence of single-dose protection, which is lowest in young children according to the only randomized, controlled trial that has been conducted to date [28]. Overall, single-dose protection was highest soon after vaccination and waned over time. These data indicate that although a single dose of killed OCV may confer a lower protection of shorter duration compared to 2 doses, it may be adequate for situations when immediate protection from cholera is needed.

This aggregated review is warranted since the killed OCVs are highly related in terms of composition. However, this study has several limitations. First, the vibriocidal assay procedures varied across the studies, raising concerns about combining and comparing results. Due to differences in methodology, the GMF rise (and related measures) may be the most practical parameter for comparison since it is a relative measure that is less affected by interlaboratory variability. Aside from differences in assay procedures, age and baseline titers in endemic and nonendemic locations may influence the immune response to OCV, as has been noted previously [29]. The relatively high proportion of variance in first-dose seroconversion explained by heterogeneity between studies as opposed to within study sampling variance, or I^2^, is likely a result of these differences between settings and laboratory methods. The blurring of the assessment of vaccine immunogenicity by prevaccination titers has also been reported for other vaccines [30]. However, we could not detect any clear patterns in the responses to the first and second dose by study location. Age group and setting may similarly affect vaccine protection estimates due to immune status and the presence or absence of ongoing natural exposure. Second, the immunogenicity data were analyzed in overlapping age distributions. Ideally, discrete age groups should have been used, but this was not possible due to data presented by varying age groups in the publications. Third, although seroepidemiological and human challenge studies have shown the association of serum antibody levels with protection [15], there is no established immunologic correlate of protection (a “protection threshold”) for OCVs. As indicated by our review, the relationship of vibriocidal antibodies to protection may be particularly uncertain in young children. The vibriocidal antibody response may be an imperfect indicator of protection, but it is currently the most commonly used marker of an immune response to OCV [15, 31]. Antibody secreting cells (ASCs) against *V. cholerae* lipopolysaccharide and O-specific polysaccharide in blood have also been detected [32, 33] but required more technically challenging procedures. Fourth, except for the Bangladesh trial [28], data on protection conferred by a single dose are from observational studies. The Bangladesh single-dose efficacy trial is therefore quite important as it confirms findings from observational studies. Fifth, protection data are based on only 6 clinical studies, with the highest estimate coming from a study in Juba, South Sudan, that measured protection within the first 2 months after vaccination [34]. In that study, the apparent protection from a single dose may have, in part, reflected boosting of immunity from natural exposure that occurred during a large epidemic the year of the study and/or the previous year. However, the immunogenicity data summarized here do suggest that protection is likely to begin within 2 weeks of the first dose, thus lending support to these short-term protection estimates. Sixth and, most important, in this review we were unable to assess exactly when protection from a single dose starts, the additional boosting that a second dose provides, or the interval between vaccine doses that maximizes the duration of protection. Except for 1 study that looked into a 28-day interval between dosing [19], no other studies that used vibriocidal antibodies assessed longer dosing intervals. In a study using ASC to measure immunogenicity, robust responses were induced after a first but not after a second dose of the bivalent OCV given 14 days later, suggesting that the current dosing schedule may not be optimal for inducing an anamnestic response [32]. To maximize the benefits of the second dose, it is critical that the interval between vaccine doses that confers the best and longest protection be established. Future studies to compare post-first and post-second dose immune kinetics may shed more light on these questions.

Protection, even if it is short term, from a single dose of OCV provides a way forward in considering alternative vaccine strategies to contain cholera outbreaks. For example, during cholera outbreaks with logistic challenges or insufficient OCV doses, single-dose coverage of a population at high risk for cholera using all immediately available vaccine could be implemented rapidly, followed by administration of a second dose when feasible [34, 35]. A second possibility, if the population at risk in an endemic area is too large to be covered, is the rapid door-to-door administration of a single dose using a ring vaccination strategy during localized outbreaks to provide protection of contacts of index cases [36]. Although it is not known how quickly vaccinees are protected, this period may be short in endemic areas with ongoing exposure to *V. cholerae*. Vaccination of secondary and tertiary contacts may prevent spread of the disease, even if the vaccine does not protect the primary contacts of the index case who have already been exposed and infected, but this assumption would need to be assessed for feasibility and effectiveness. The second dose could potentially be self-administered or deployed through vaccination posts at a later date [37].

When considering the use of single-dose OCV, it should be kept in mind that the metaanalysis of the immunogenicity data and the subgroup analysis of the Bangladesh study showed lower immune response and inadequate protection among children aged <5 years. Based on principles of cocooning [38], oral cholera vaccination of older children and adults around those too young to be vaccinated or to mount an adequate response could be beneficial. While both 1 and 2 doses of killed OCV appears to be less protective for young children, there is evidence for substantial indirect protection for these children when a large proportion of older persons in the community are vaccinated [39].

## Supplementary Data

Supplementary materials are available at *Clinical Infectious Diseases* online. Consisting of data provided by the authors to benefit the reader, the posted materials are not copyedited and are the sole responsibility of the authors, so questions or comments should be addressed to the corresponding author.

Supplementary Data

## Notes

***Author contributions.*** A. L. L. and J. D. conducted the literature search, compiled and screened the list of articles, selected the relevant documents, reviewed the eligible articles, and extracted the data. All authors provided additional data sources. A. L. L., J. D., and A. S. A. analyzed the information and interpreted the data. A. L. L., J. D., and A. S.A. wrote the initial draft. J. D. and A. S. A. prepared the figures. All authors contributed to the writing and revision of the text and finalization of the manuscript.

**Disclaimer.** The funders had no role in study design, data collection and analysis, decision to publish, or preparation of the manuscript.

***Financial support.*** This work was supported by the Delivering Oral Vaccine Effectively project through a grant from the Bill and Melinda Gates Foundation (OPP1053556) administered by the Johns Hopkins Bloomberg School of Public Health.

***Potential conflicts of interest.*** All authors: No reported conflicts of interest. All authors have submitted the ICMJE Form for Disclosure of Potential Conflicts of Interest. Conflicts that the editors consider relevant to the content of the manuscript have been disclosed.
