# Supplementary material for: Immunogenicity and Protection From a Single Dose of Internationally Available Killed Oral Cholera Vaccine: A Systematic Review and Metaanalysis
Source: Clin Infect Dis. 2017 Nov 21;66(12):1960–71. doi: 10.1093/cid/cix1039 (PMC5982790; doi:10.1093/cid/cix1039)
Supplement: Supplementary Data [file cix1039_suppl_supplementary_data.docx]

Supplementary data to: Lopez AL, Deen J, Azman AS, et al. Immunogenicity and protection from a single dose of internationally available, killed oral cholera vaccine: a systematic review and meta-analysis

**Supplementary Table 1: Details and grading of studies included in the systematic review**

| **Study (Reference number in manuscript)** | **Country** | **Study period** | **Study design** | **Intervention** | **Study population n.** | **Age** | **Risk of bias within the study [**[**1**](#_ENREF_1)**] (main biases)** | **Quality grading [**[**2**](#_ENREF_2)**]*** |
| --- | --- | --- | --- | --- | --- | --- | --- | --- |
| **Single-dose vaccine immunogenicity studies** | | | | | | | | |
| Baik, 2014 [[3](#_ENREF_3)] | Korea | 24 September 2012 to 7 February 2013 | Phase 1 open label | Euvichol | 22 | Adults | Moderate (open label study, all healthy adult male volunteers with age of at least 20 yrs) | Moderate |
| Baik, 2015 [[4](#_ENREF_4)] | Philippines | May to September 2014 | Phase 2 Randomized, non-inferiority, controlled trial | Euvichol vs. Shanchol | 777  486 | Adults  Children | Low (Bias unlikely due to study design) | High |
| Kanungo, 2015 [[5](#_ENREF_5)] | Kolkata, India | January 2010 to October 2011 | Phase 2, double-blind, randomized, non-inferiority, placebo-controlled trial | Shanchol | 178  178 | Adults  Children | Low (Bias unlikely due to study design) | High |
| Kanungo, 2015 [[6](#_ENREF_6)] | Kolkata, India | May-June 2012 | Nested Open Label Non-inferiority Trial | Shanchol | 187  183 | ≥15 years  6-14 years | Low (Bias unlikely due to earlier randomization in the original study) | Moderate |
| Kanungo, 2009 [[7](#_ENREF_7)] | Kolkata, India | June to August 2007 | Phase 2, randomized, non-inferiority, placebo-controlled trial | Shanchol | 77  77 | Adults  Children | Low (Bias unlikely due to study design) | High |
| Desai, 2015 [[8](#_ENREF_8)] | Ethiopia | December 2012 to July 2013 | Randomized, placebo-controlled trial | Shanchol | 106  106 | Adults  Children | Low (Bias unlikely due to study design) | High |
| Ivers, 2015 [[9](#_ENREF_9)] | Haiti | April 2013 | Open label | Shanchol | 50 | Adults | Moderate (open label, potential selection and detection bias) | Moderate |
| Saha, 2011 [[10](#_ENREF_10)] | Bangladesh | 2010 | Double-blind placebo-controlled trial | Shanchol | 110  110  110 | Adults  Toddlers  Younger children | Low (Bias unlikely due to study design) | High |
| Charles, 2014 [[11](#_ENREF_11)] | Haiti | April 2013 | Open label | Shanchol | 25  51  47 | Adults  6-17 years  1-5 years | Moderate (open label, potential selection and detection bias) | Moderate |
| Alam, 2011 [[12](#_ENREF_12)] | Bangladesh | October 2008 and June 2010 | Open label | Dukoral | 60 | 18-45 years | Moderate (open label, potential selection and detection bias) | Moderate |
| Ahmed, 2009 [[13](#_ENREF_13)] | Bangladesh | June 2005 and May 2007 | Open label | Dukoral | 176  164 | 10-18 months  6-9 months | Moderate (open label, potential selection and detection bias) | Moderate |
| Saha, 2016 [[14](#_ENREF_14)] | Bangladesh | June 2012 to December 2015 | Open label | Shanchol | 580 | 18-45 years | Moderate (open label, potential selection and detection bias) | Moderate |
| Iyer, 2016 [[15](#_ENREF_15)] | South Sudan | June 2015 | Open label | Shanchol | 101  67  37 | ≥18 years  6-17 years  1-5 years | Moderate (open label, potential selection and detection bias) | Moderate |
| Leung, 2012 [[16](#_ENREF_16)] | Bangladesh | Not stated | Open label | Dukoral | 20  20 | 6-17 years  2-5 years | Moderate (open label, potential selection and detection bias) | Moderate |
| Matias, 2016 [[17](#_ENREF_17)] | Haiti | May 2015 | Open label | Shanchol | 24 | 18-60 years | Moderate (open label, potential selection and detection bias) | Moderate |
| Alam, 2013 [[18](#_ENREF_18)] | Bangladesh | October 2008 and June 2010 | Open label | Dukoral | 33  20  20 | 18-45 years  6-17 years  2-5 years | Moderate (open label, potential selection and detection bias) | Moderate |
| Aloysia, 2015 [[19](#_ENREF_19)] | Philippines | March 2014 to February 2015 | Open label | Shanchol | 112  112  112 | ≥15 years  5 – 14 years  1-4 years | Moderate (Potential bias, information available from presentation incomplete) | Moderate |
| **Single-dose vaccine protection studies** | | | | | | | | |
| Wierzba, 2015 [[20](#_ENREF_20)] | Odisha, India | 2011 | Test-negative design | Shanchol | - 1 dose: 31,552 - 2 doses: 23,751 | 1 year and older | Moderate (Potential effects of healthcare seeking behavior on vaccine effectiveness measurement minimized by test-negative design). | Moderate |
| Ivers, 2015 [[21](#_ENREF_21)] | Haiti | 2012 | Case-control study | Shanchol | - 45,417 persons received at least one dose and 91% of them received a second dose | 1 year and older | Moderate (Potential bias related to healthcare seeking behavior or ascertainment of vaccination assessed using a bias-indicator component). | Moderate |
| Luquero, 2014 [[22](#_ENREF_22)] | Guinea | 2012 | Case-control study | Shanchol | - 1 dose: 172,544 - 2 doses: 143,706 | Older than 12 months | Moderate (Potential bias related to healthcare seeking behavior or ascertainment of vaccination assessed using a bias-indicator component). | Moderate |
| Qadri, 2016 [[23](#_ENREF_23)] | Bangladesh | 2013 | Phase III randomised control trial | Shanchol | - 102,552 single-dose recipients - 102,148 placebo recipients | 1 year and older | Low (Bias unlikely due to study design). | High |
| Khatib, 2012 [[24](#_ENREF_24)] | Zanzibar | 2008 | Cohort study | Dukoral | - No dose: 20,500 - 1 dose: 3,757 - 2 doses: 23,921 | 2 years and older | Moderate (Potential bias related to healthcare seeking behavior or ascertainment of vaccination assessed using a bias-indicator component). | Moderate |
| Azman, 2016 [[25](#_ENREF_25)] | Juba, South Sudan | 2015 | Case-cohort study | Shanchol | - 165,000 single dose recipients | 1 year and older | Moderate (Potential bias related to healthcare seeking behavior or ascertainment of vaccination assessed using a bias-indicator component). | Moderate |

***Definitions of grading scores[**[**2**](#_ENREF_2)**]:**

- High – We are very confident that the true effect lies close to that of the estimate of the effect.
- Moderate – We are moderately confident in the effect estimate: The true effect is likely to be close to the estimate of the effect, but there is a possibility that it is substantially different.
- Low – Our confidence in the effect estimate is limited: The true effect may be substantially different from the estimate of the effect.
- Very low – We have very little confidence in the effect estimate: The true effect is likely to be substantially different from the estimate of effect.

**References**

1. Guyatt GH, Oxman AD, Vist GE, et al. GRADE guidelines: 4. Rating the quality of evidence -- study limitations (risk of bias). Journal of clinical epidemiology **2011**; 64: 407-15.

2. Balshem H, Helfand M, Schunemann HJ, et al. GRADE guidelines: 3. Rating the quality of evidence. Journal of clinical epidemiology **2011**; 64: 401-6.

3. Baik YO, Choi SK, Kim JW, et al. Safety and immunogenicity assessment of an oral cholera vaccine through phase I clinical trial in Korea. Journal of Korean medical science **2014**; 29(4): 494-501.

4. Baik YO, Choi SK, Olveda RM, et al. A randomized, non-inferiority trial comparing two bivalent killed, whole cell, oral cholera vaccines (Euvichol vs Shanchol) in the Philippines. Vaccine **2015**; 33(46): 6360-5.

5. Kanungo S, Desai SN, Nandy RK, et al. Flexibility of oral cholera vaccine dosing-a randomized controlled trial measuring immune responses following alternative vaccination schedules in a cholera hyper-endemic zone. PLoS neglected tropical diseases **2015**; 9(3): e0003574.

6. Kanungo S, Desai SN, Saha J, et al. An Open Label Non-inferiority Trial Assessing Vibriocidal Response of a Killed Bivalent Oral Cholera Vaccine Regimen following a Five Year Interval in Kolkata, India. PLoS neglected tropical diseases **2015**; 9(5): e0003809.

7. Kanungo S, Paisley A, Lopez AL, et al. Immune responses following one and two doses of the reformulated, bivalent, killed, whole-cell, oral cholera vaccine among adults and children in Kolkata, India: a randomized, placebo-controlled trial. Vaccine **2009**; 27(49): 6887-93.

8. Desai SN, Akalu Z, Teshome S, et al. A Randomized, Placebo-Controlled Trial Evaluating Safety and Immunogenicity of the Killed, Bivalent, Whole-Cell Oral Cholera Vaccine in Ethiopia. Am J Trop Med Hyg **2015**; 93(3): 527-33.

9. Ivers LC, Charles RC, Hilaire IJ, et al. Immunogenicity of the Bivalent Oral Cholera Vaccine Shanchol in Haitian Adults With HIV Infection. J Infect Dis **2015**; 212(5): 779-83.

10. Saha A, Chowdhury MI, Khanam F, et al. Safety and immunogenicity study of a killed bivalent (O1 and O139) whole-cell oral cholera vaccine Shanchol, in Bangladeshi adults and children as young as 1 year of age. Vaccine **2011**; 29(46): 8285-92.

11. Charles RC, Hilaire IJ, Mayo-Smith LM, et al. Immunogenicity of a killed bivalent (O1 and O139) whole cell oral cholera vaccine, Shanchol, in Haiti. PLoS neglected tropical diseases **2014**; 8(5): e2828.

12. Alam MM, Riyadh MA, Fatema K, et al. Antigen-specific memory B-cell responses in Bangladeshi adults after one- or two-dose oral killed cholera vaccination and comparison with responses in patients with naturally acquired cholera. Clinical and vaccine immunology : CVI **2011**; 18(5): 844-50.

13. Ahmed T, Svennerholm AM, Al Tarique A, Sultana GN, Qadri F. Enhanced immunogenicity of an oral inactivated cholera vaccine in infants in Bangladesh obtained by zinc supplementation and by temporary withholding breast-feeding. Vaccine **2009**; 27(9): 1433-9.

14. Saha A, Khan A, Salma U, et al. The oral cholera vaccine Shanchol when stored at elevated temperatures maintains the safety and immunogenicity profile in Bangladeshi participants. Vaccine **2016**; 34(13): 1551-8.

15. Iyer AS, Bouhenia M, Rumunu J, et al. Immune Responses to an Oral Cholera Vaccine in Internally Displaced Persons in South Sudan. Nature Sci rep **2016**; 6: 35742.

16. Leung DT, Rahman MA, Mohasin M, et al. Memory B cell and other immune responses in children receiving two doses of an oral killed cholera vaccine compared to responses following natural cholera infection in Bangladesh. Clinical and vaccine immunology : CVI **2012**; 19(5): 690-8.

17. Matias WR, Falkard B, Charles RC, et al. Antibody Secreting Cell Responses following Vaccination with Bivalent Oral Cholera Vaccine among Haitian Adults. PLoS neglected tropical diseases **2016**; 10(6): e0004753.

18. Alam MM, Leung DT, Akhtar M, et al. Antibody avidity in humoral immune responses in Bangladeshi children and adults following administration of an oral killed cholera vaccine. Clinical and vaccine immunology : CVI **2013**; 20(10): 1541-8.

19. Aloysia N, Thollot Y, Capeding MR, Gonzales MLA, Rasuli A, Dhingra M. Safety and immunogenicity of the killed bivalent (O1 and O139) whole-cell oral cholera vaccine in the Philippines: Results of the first Asian study outside the Indian subcontinent. In: Vaccines for Enteric Diseases. Edinburgh, UK, 2015.

20. Wierzba TF, Kar SK, Mogasale VV, et al. Effectiveness of an oral cholera vaccine campaign to prevent clinically-significant cholera in Odisha State, India. Vaccine **2015**; 33(21): 2463-9.

21. Ivers LC, Hilaire IJ, Teng JE, et al. Effectiveness of reactive oral cholera vaccination in rural Haiti: a case-control study and bias-indicator analysis. The Lancet Global health **2015**; 3(3): e162-8.

22. Luquero FJ, Grout L, Ciglenecki I, et al. Use of Vibrio cholerae vaccine in an outbreak in Guinea. N Engl J Med **2014**; 370(22): 2111-20.

23. Qadri F, Wierzba TF, Ali M, et al. Efficacy of a Single-Dose, Inactivated Oral Cholera Vaccine in Bangladesh. N Engl J Med **2016**; 374(18): 1723-32.

24. Khatib AM, Ali M, von Seidlein L, et al. Effectiveness of an oral cholera vaccine in Zanzibar: findings from a mass vaccination campaign and observational cohort study. The Lancet Infectious diseases **2012**; 12(11): 837-44.

25. Azman AS, Parker LA, Rumunu J, et al. Effectiveness of one dose of oral cholera vaccine in response to an outbreak: a case-cohort study. The Lancet Global health **2016**; 4(11): e856-e63.

**Supplementary Figure 1: Search strategy**

The following search strings were used to search all fields in MEDLINE through PubMed [search conducted on 14 November 2016]:

| ("killed cholera vaccine" OR "oral cholera vaccine" OR OCV) AND (immun* OR serolog* OR "immune response" OR "serologic response" OR vibriocidal AND (("2005/01/01"[PDAT] : "2016/11/13"[PDAT]))) | Results = 52 articles |
| --- | --- |
| (“killed cholera vaccine” OR "oral cholera vaccine" OR OCV) AND (protect* OR efficac* OR effective* AND (("2005/01/01"[PDAT] : "2016/11/13"[PDAT]))) | Results = 108 articles |

The following search strings were used to search all text in the Cochrane Central Register of Controlled Trials limited to the years 2005 to 2016 [search conducted on 14 November 2016]

| oral near cholera near vaccine not live AND (immun* OR serolog* OR immune response OR serologic response OR vibriocidal) | Results = 33 articles |
| --- | --- |
| oral near cholera near vaccine not live AND (protect* OR efficac* OR effective*) | Results = 25 articles |

The following search strings were used to search title, abstract, keywords in Scopus limited to the years 2005 to 2016 [search conducted on 14 November 2016]:

| ("killed cholera vaccine" OR "oral cholera vaccine" OR OCV) AND (immun* OR serolog* OR "immune response" OR "serologic response" OR vibriocidal)   - Exclude subject area: veterinary - Exclude document type: review, note, short survey, erratum, letter, book chapter, editorial | Results = 110 articles |
| --- | --- |
| (“killed cholera vaccine” OR "oral cholera vaccine" OR OCV) AND (protect* OR efficac* OR effective*)   - Exclude subject area: veterinary - Exclude document type: review, note, short survey, erratum, letter, book chapter, editorial | Results = 93 articles |

Total number of records from the databases downloaded into Endnote = 421

**Supplementary Figure 2: Seroconversion to the Inaba serotype after the first and second dose of a bivalent oral cholera vaccine in young children, children, and adults**


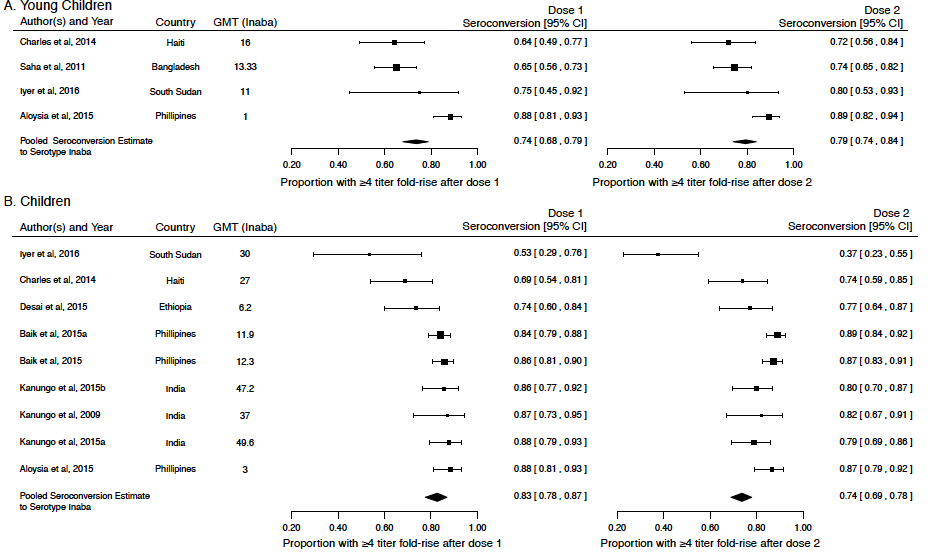


**
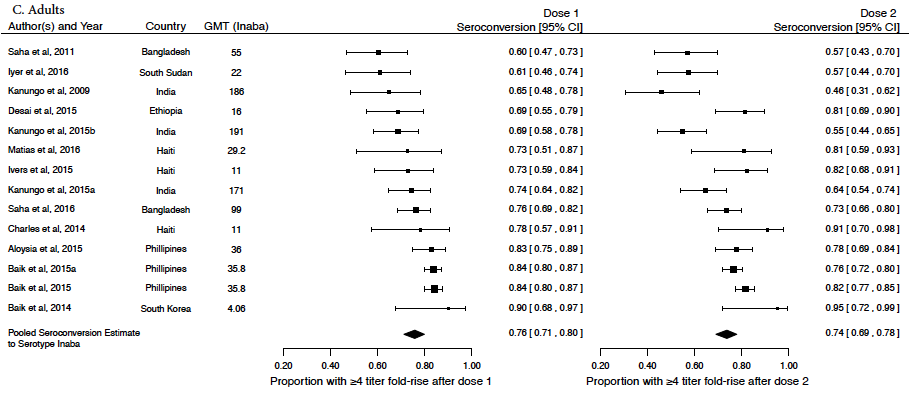
**
